# Genetic Structure, Nestmate Recognition and Behaviour of Two Cryptic Species of the Invasive Big-Headed Ant *Pheidole megacephala*

**DOI:** 10.1371/journal.pone.0031480

**Published:** 2012-02-21

**Authors:** Denis Fournier, Maurice Tindo, Martin Kenne, Paul Serge Mbenoun Masse, Vanessa Van Bossche, Eliane De Coninck, Serge Aron

**Affiliations:** 1 Evolutionary Biology and Ecology, Université Libre de Bruxelles, Brussels, Belgium; 2 Département de Biologie des Organismes Animaux, Faculté des Sciences, Université de Douala, Douala, Cameroun; 3 Laboratoire de Zoologie, Faculté des Sciences, Université de Yaoundé I, Yaoundé, Cameroun; 4 Royal Museum for Central Africa, Tervuren, Belgium; University of Western Ontario, Canada

## Abstract

**Background:**

Biological invasions are recognized as a major cause of biodiversity decline and have considerable impact on the economy and human health. The African big-headed ant *Pheidole megacephala* is considered one of the world's most harmful invasive species.

**Methodology/Principal Findings:**

To better understand its ecological and demographic features, we combined behavioural (aggression tests), chemical (quantitative and qualitative analyses of cuticular lipids) and genetic (mitochondrial divergence and polymorphism of DNA microsatellite markers) data obtained for eight populations in Cameroon. Molecular data revealed two cryptic species of *P. megacephala*, one inhabiting urban areas and the other rainforests. Urban populations belong to the same phylogenetic group than those introduced in Australia and in other parts of the world. Behavioural analyses show that the eight populations sampled make up four mutually aggressive supercolonies. The maximum distance between nests from the same supercolony was 49 km and the closest distance between two nests belonging to two different supercolonies was 46 m. The genetic data and chemical analyses confirmed the behavioural tests as all of the nests were correctly assigned to their supercolony. Genetic diversity appears significantly greater in Africa than in introduced populations in Australia; by contrast, urban and Australian populations are characterized by a higher chemical diversity than rainforest ones.

**Conclusions/Significance:**

Overall, our study shows that populations of *P. megacephala* in Cameroon adopt a unicolonial social structure, like invasive populations in Australia. However, the size of the supercolonies appears several orders of magnitude smaller in Africa. This implies competition between African supercolonies and explains why they persist over evolutionary time scales.

## Introduction

The recent increase in human activity and commerce has, intentionally or unintentionally, greatly contributed to the dispersal of exotic species and their introduction into new habitats. In most cases, introduced species do not survive and, among those introduced species that do, many do not cause problems of any sort. However, some species' overwhelming invasive success upsets the balance in the invaded ecosystems and constitutes an important threat to biodiversity [1]. These biological invaders have a considerable impact on the economy and on public health [2].

Biological invaders represent a paradox for evolutionary biologists [3]. Introduced populations usually suffer a dramatic reduction in genetic diversity due to bottleneck effects and genetic drift. They may experience inbreeding depression leading to more recessive, deleterious traits. Often, they have slow population growth because low densities lead to a failure to mate (*i.e.* the Allee effect [4]). Yet, in their introduced range biological invaders (which, by definition, are present in biogeographical regions where they did not evolve) frequently triumph in the competition for ecological resources over native populations that are shaped by natural selection to fit their environment and local conditions. A rich and expanding body of literature has been produced to help to understand why some species become successful invaders and to decipher the processes involved in biological invasions [5], [6], [7], [8], [9]. It ensues that demographic, ecological and evolutionary factors, associated both with the invader and with the environment, jointly influence the success of an invasion [10], [11], [12]. Migratory ability, freedom from natural enemies, biotic or abiotic changes in a given area, the phylogeographical history of the invader, phenotypical adaptive traits, and hybridization events occurring in the new environment can shape the interaction between the invader and the invaded environment.

Ants are among the most ecologically successful groups of organisms [13]. About 150 ant species (of the 12,642 species described to date, http://antbase.org/ – December 2011) have successfully established populations outside their native range [14]; many have caused major economic losses as well as changes in both species composition and the functioning of ecosystems [15]. Some life-history traits may have promoted their success as invaders: the world's five most invasive ant species are all omnivorous, they have adopted an opportunistic nesting behaviour, they are found living in human-disturbed environments, their nests have a large number of reproductive queens (polygyny), and they show exacerbated aggressiveness towards other ant species but a reduced intraspecific aggressiveness at the population level [16], [17], [18], [19]. In ants and other social insects, individuals discriminate colony members (nestmates) from foreign ones (non-nestmates) through multicomponent chemical signatures present on the surface of the cuticle [20], [21], [22]. The lack of intraspecific aggressiveness results in the formation of supercolonies where nests have no clear boundaries, and workers, brood and queens move freely between the nests. Supercolonies are made up of such a large number of nest units that individuals from distant nests are unlikely to come into direct contact with one another [23]. Populations may then be composed of the assemblage of supercolonies co-existing in close proximity or, at the endpoint of a *continuum*, they are unicolonial and consist of a single, huge supercolony.

The evolutionary forces leading to unicoloniality may be diverse [24], [25], [26], but their ecological consequences are similar: unicoloniality eliminates the costs of intra-specific competition and combines the worker forces of different nests to conquer new territories. Moreover, polygyny increases the fertility of the nests. The combination of unicoloniality and polygyny provides a decisive advantage to invasive ants because it results in extremely high population densities and allows them to efficiently monopolise environmental resources [27]. Recent findings have, however, challenged the role of unicoloniality in the invasive success of introduced populations by pointing out the absence of clear colonial boundaries leading to the formation of supercolonies and/or unicolonial populations in the native range of two invasive ants, the Argentine ant *Linepithema humile* [23], [24] and the little fire ant *Wasmannia auropunctata* [28]. Thus, unicoloniality is not necessarily a derived trait that evolves after introduction, and other demographic, ecological or evolutionary factors could explain the ecological dominance of invasive populations.

The African big-headed ant *Pheidole megacephala* forms a complex including at least ten subspecies, all described in Sub-Saharan Africa [29], [30]. According to their geographic distribution, Wheeler [29] hypothesized an Ethiopian or Malagasy origin of the species; but this remains debated [31]. This extremely widespread and destructive species is listed as one of the 100 worst invasive organisms by the International Union for the Conservation of Nature (IUCN) [32]. *P. megacephala* is dominant in the many areas it has invaded [31]. In Australia, it has been shown that threatens native biodiversity and natural ecosystems, infests houses, and negatively impacts horticultural and agricultural production [33]. The species' ecological success can be linked to its unicolonial social structure. Workers are not aggressive towards conspecifics from different nests, even over large geographical scales (up to 3000 km) and between populations encompassing a wide range of environmental conditions [34]. The lack of aggressiveness is associated with the absence of genetic differentiation between nests, and reduced chemical differentiation between populations [34]. While the consequences of its invasive success and the life history traits of the African big-headed ant *P. megacephala* have been widely documented in the Pacific [31], [34], [35], studies on the species in Africa are scarce [36], [37] and no data are available concerning its population genetics and social structure.

**Figure 1:**
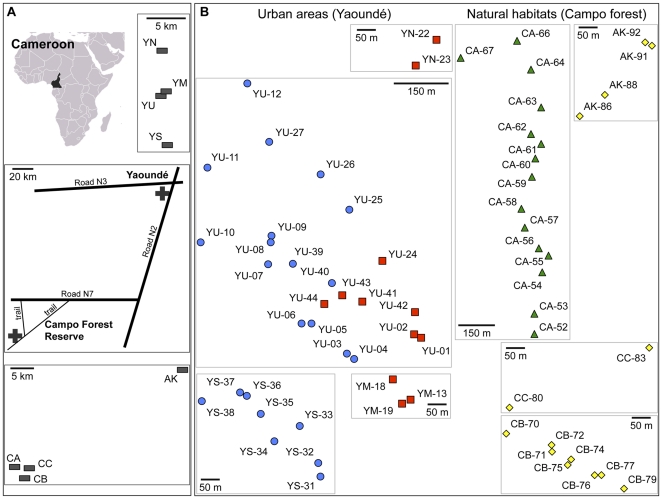
Study site and nest locations. A, Locations of the two ecological zones (+) and of the eight populations collected (−). YM: Ministère de la Recherche Scientifique et de l'Innovation; YN: North of Yaoundé; YS: South of Yaoundé; YU: Université de Yaoundé I; CA, CB and CC: populations A, B and C in the Campo Forest Reserve; AK: Akok. B, Locations of the nests within each of the eight populations. Populations YM, YN, YS and YU correspond to *P. megacephala* var. 1 and populations AK, CA, CB and CC to *P. megacephala* var. 2. Nests belonging to the same supercolony (cf. Results section) are indicated by the same symbol.

We adopted a pluralistic approach to investigate populations of *P. megacephala* in Cameroon, west Central Africa (Figure 1). First, we used mtDNA sequence variation to assess phylogenetic relationships between individuals from Cameroon and those previously sampled in invaded areas. Second, by using DNA microsatellite loci, we studied the genetic diversity and genetic differentiation of Cameroonian populations 0.8–230 km apart. Third, our genetic data were supplemented by worker aggression tests between individuals from different nests and populations to determine colony boundaries. Fourth, we conducted gas chromatography and mass spectrometry (GC-MS) analyses to make qualitative and quantitative comparisons of the cuticular profiles within and between populations. Finally, we tested for a possible correlation between behavioural, spatial, chemical and genetic data. In addition, we gauged the contribution of the different levels of population structure to patterns of chemical and genetic variation. Our results are compared with those previously reported for *P. megacephala* in South-Africa and Australia to investigate possible evolutionary changes.

## Results

### Phylogenetic relationships

**Figure 2:**
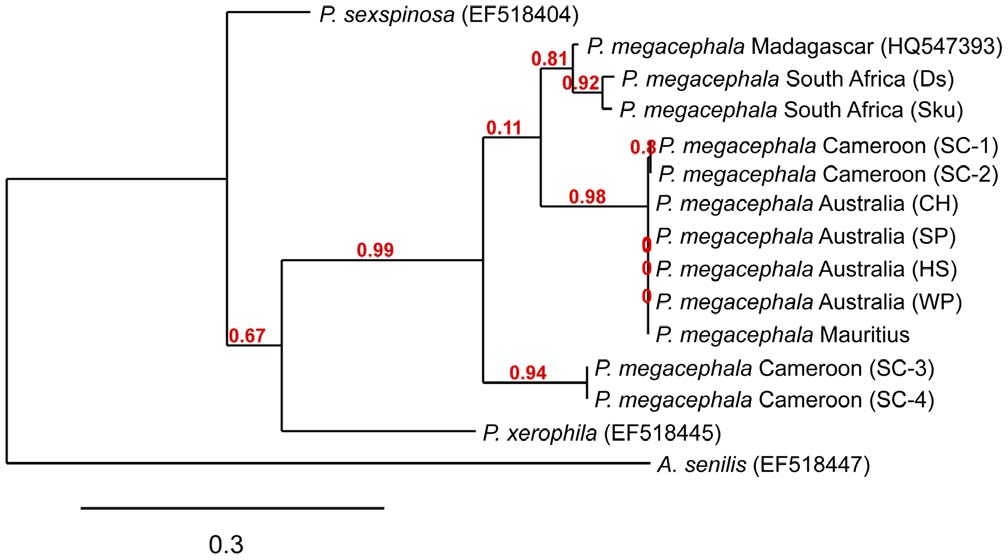
Neighbour-joining tree of *Pheidole megacephala* from different localities, the sister species *Pheidole sexspinosa* and *P. xerophila*, and the outgroup *Aphaenogaster senilis* based on COI. The percentage bootstrap supports are shown above the branches for the major groups. GeneBank accession numbers, population or supercolonies are indicated between brackets. Australian, South African and Malagasy samples refer to [34], [40] and [39], respectively. Mauritian samples come from personal collection (DF). Sequences for *P. sexspinosa*, *P. xerophyla* and *Aphaenogaster senili*s are taken from [38].

The phylogenetic tree reconstructed on the basis of mtDNA sequences (Figure 2) reveals that Cameroonian populations of *P. megacephala* form two phylogenetic groups (*P. megacephala* var. 1 and *P. megacephala* var. 2) that evolved independently from a very long time. These two phylogenetic groups exactly match with the two ecological zones studied, *P. megacephala* var. 1 corresponding to the specimens collected in urban areas and *P. megacephala* var. 2 to those sampled in rainforest. The position of *P. sexspinosa* and *P. xerophila* on the phylogenetic tree, two sister species of *P. megacephala* [38], indicates that the two phylogenetic groups identified correspond to two cryptic species of the African big-headed ant. Moreover, our genetic analyses show that mtDNA sequences of *P. megacephala* var. 1 (*i.e.* urban samples) and of Australian specimens belong to the same phylogenetic group. They are also very close to those isolated from samples collected in Mauritius ([39]; DF, personal data), South Africa [40] and Madagascar [41] (sequences and locations were retrieved by crossing databases available at http://www.antweb.org/ and http://www.boldsystems.org/). Furthermore, the Basic Local Alignment Search Tool (BLAST) shows high homologies between sequences obtained from *P. megacephala* var. 1 and sequences published for samples collected in Seychelles (*E*-values very close to zero), and BLAST for *P. megacephala* var. 2 gave the highest scores with samples collected in Gabon and Comoros (http://www.antweb.org/ and http://www.boldsystems.org/).

### Morphological studies

The size of individuals estimated by the maximum head width was different between the two cryptic species. *Minor* were lower in *P. megacephala* var. 1 (urban) than in *P. megacephala* var. 2 (rainforest) populations (mean ± se = 0.617±0.009 and 0.672±0.011, respectively; Mann-Whitney test, *p*<0.001). Morphological observations at electron microscope also show that *minor* of *P. megacephala* var. 1 have shorter spines on the propodeum than *minor* of *P. megacephala* var. 2 (see Supplementary materials, Figures S1 and S2). In addition, hairs on the petiole of *P. megacephala* var. 1 end in a point, whereas they form a brush in *P. megacephala* var. 2 (see Supplementary materials, Figure S3).

### Behavioural assays

**Figure 3:**
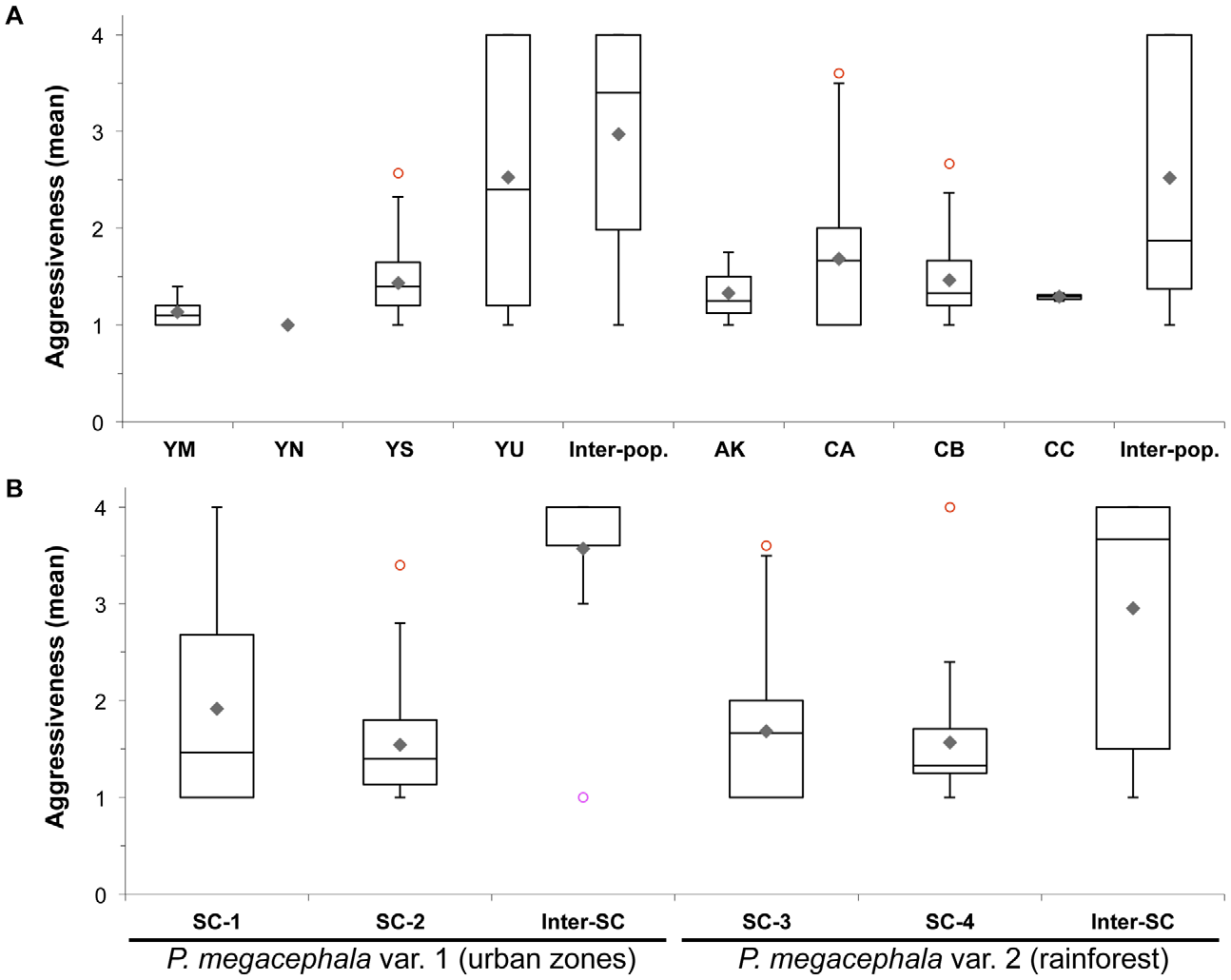
Average intra- and inter-group aggressiveness between *P. megacephala* workers. Box-plots indicate median (horizontal line), mean (diamond), interquartile range (box) and minimum and maximum values (whiskers). Nests are assigned to their population (A) or supercolony (B). (AK: Akok; CA, CB and CC: Campo Forest Reserve; YM, YN, YS and YU: Yaoundé and its periphery).

No aggressive behaviour was observed in control experiments involving two workers from the same nest (1.067±0.064, min-max 1–2, *n* = 15 tests). Behavioural assays testing the responses of two *major* or two *minor* from different nests yielded consistent results (*n* = 39 tests, Pearson's correlation, *r_P_* = 0.905, *p*<0.001). By contrast, encounters between individuals from different populations resulted in aggressive responses in 60.10% of the 386 trials (*P. megacephala* var. 1: mean worker aggressiveness ± se = 2.947±0.070; *P. megacephala* var. 2: 1.933±0.188) (Figure 3A). Agonistic interactions were also observed, though to a lesser extent, between workers originating from different nests in five out of the eight populations sampled. In populations of *P. megacephala* var. 1, 46.12% of the responses were aggressive in YU (*n* = 722 trials; mean worker aggressiveness ± se = 2.526±0.109) and 3.38% in YS (*n* = 148; 1.432±0.068); in populations of *P. megacephala* var. 2, 9.09% of aggressive interactions was observed in AK (*n* = 11; 1.333±0.220), 12.94% in CA (*n* = 201; 1.687±0.089) and 7.14% in CB (*n* = 56; 1.468±0.116) (Figure 3A). However, no aggressiveness occurred within populations YM (*n* = 30 trials; 1.133±0.067) and YN (*n* = 5; 1) of *P. megacephala* var. 1 or CC (*n* = 7; 1.292±0.042) of *P. megacephala* var. 2 (Figure 3A).

Our behavioural data suggested the existence of supercolonies in each of the two cryptic species of *P. megacephala*. Two supercolonies, SC-1 and SC-2, cohabit in population YU where workers displayed aggressive behaviours in roughly one test out of two (Figures 1 and 3A). Nests from populations YM and YN could be assigned to supercolony SC-1, and those from population YS to supercolony SC-2. Supercolony SC-3 corresponds to the nests of population CA and supercolony SC-4 groups the nests of populations AK, CB and CC (Figure 1). Consistent with this nest distribution, in both cryptic species workers from the same supercolony tolerated each other (mean worker aggressiveness ± se = 1.660±0.048), whereas workers from different supercolonies were aggressive towards each other (3.489±0.092; Mann-Whitney test, p<0.001) (Figure 3B). In the latter situation, both opponents acted aggressively and fought until the death of one of the antagonists.

### Genetic diversity, relatedness and population structure

One hundred and eight alleles over eight microsatellites loci were found across all of the eight populations sampled. Thirty-three alleles were detected exclusively in *P. megacephala* var. 1 populations (*i.e.* urban areas) and 52 in *P. megacephala* var. 2 populations (*i.e.* rainforest); 23 alleles were found in both species. Measurements of genetic diversity computed for *P. megacephala* var. 2 (number of alleles per locus *A* = 5.563±0.568, allelic richness based on the smallest sample size (*N* = 22) *Ar*_[22]_ = 4.233±0.306 and genetic diversity *H* = 0.609±0.033) were higher than for *P. megacephala* var. 1 (*A* = 4.333±0.393, *Ar*_[22]_ = 3.330±0.236 and *H* = 0.548±0.036). The inbreeding coefficient computed for *P. megacephala* var. 2 (*f* = 0.097±0.053) was lower than for *P. megacephala* var. 1 (*f* = 0.120±0.067) (Table 1). The allelic richness was significantly different between the two groups (*p* = 0.011); however, they did not differ by their number of alleles, their genetic diversity or their inbreeding coefficient (*p* = 0.065, 0.063 and 0.627, respectively).

**Table 1:** Number of nests sampled, location and descriptive population statistics (mean ± se and [95% confidence interval]) for genetic and chemical analyses of eight Cameroonian populations of the invasive ant *P. megacephala*.

	Genetic data						Chemical data	
	*A*	*Ar*	*H*	*f*	*r*	*Fst*	*I*	*E*
*P. megacephala* var. 1								
(urban populations)								
YM (3 nests)	3.571±0.429	3.296±0.439	0.533±0.059	0.075±0.090	0.746±0.102	0.134±0.061	-	-
N3.86053 E11.50730				[−0.146–0.296]	[0.306–1.187]	[−0.126;0.394]		
YN (2 nests)	4.250±0.675	3.679±0.498	0.602±0.073	−0.031±0.079	0.397±0.104	0.088	0.949±0.002	0.082
N3.89885 E11.49714				[−0.219–0.156]	[−0.930–1.724]			
YS (8 nests)	3.143±0.634	2.368±0.343	0.416±0.094	0.025±0.207	0.792±0.038	0.144±0.020	0.944±0.004	0.111±0.007
N3.79724 E11.49942				[−0.480–0.531]	[0.701–0.881]	[0.102–0.186]	0.944±0.004	[0.096–0.126]
YU (22 nests)	6.125±0.895	3.851±0.463	0.622±0.042	0.392±0.096	0.656±0.035	0.366±0.003	0.927±0.013	0.168±0.010
N3.85707 E11.50323				[0.166–0.618]	[0.582–0.730]	[0.359–0.372]		[0.148–0.188]
Overall	4.333±0.393^a^	3.330±0.236^a^	0.548±0.036^a^	0.120±0.067^a^	0.680±0.029^a^	0.247±0.010^a^	0.933±0.010^a^	0.151±0.006^a^
				[−0.016–0.256	[0.620–0.739]	[0.227–0.266]		[0.140–0.162]
*P. megacephala* var. 2								
(rainforest populations)								
AK (4 nests)	4.000±0.802	3.512±0.557	0.597±0.042	−0.103±0.161	0.711±0.052	0.202±0.045	0.836±0.036	0.456±0.026
N2.78759 E10.27990				[−0.485–0.279]	[0.545–0.877]	[0.087–0.316]		[0.402–0.508]
CA (15 nests)	7.125±1.469	4.465±0.684	0.560±0.084	0.190±0.085	0.721±0.026	0.216±0.006	0.705±0.019	0.364±0.017
N2.60206 E9.87888				[−0.010–0.390]	[0.665–0.776]	[0.203–0.227]		[0.330–0.398]
CB (8 nests)	7.125±1.156	5.039±0.719	0.640±0.056	0.231±0.041	0.688±0.053	0.242±0.026	0.773±0.027	0.472±0.022
N2.57509 E9.89992				[0.134–0.328]	[0.563–0.814]	[0.188–0.295]		[0.429–0.514]
CC (2 nests)	4.000±0.423	3.914±0.419	0.640±0.083	0.070±0.073	0.556±0.075	-	0.801±0.026	0.421±0.031
N2.60047 E9.92201				[−0.102–0.241]	[0.393–0.506]			[0.356–0.488]
Overall	5.563±0.568^a^	4.233±0.306^b^	0.609±0.033^a^	0.097±0.053^a^	0.699±0.022^a^	0.220±0.007^a^	0.765±0.015^b^	0.444±0.006^b^
				[−0.011–0.205	[0.654–0.744]	[0.206–0.234]		[0.432–0.455]
Overall	13.500±2.228†	13.271±2.210†	0.756±0.036†	0.108±0.075	0.689±0.019	0.237±0.007	0.837±0.013	0.236±0.010
				[0.023–0.191]	[0.651–0.726]	[0.223–0.251]		[0.217–0.256]

† Mean ± se population genetic statistics over eight loci. *A*: number of alleles; *Ar*: allelic richness; *H*: gene diversity; *f*: inbreeding coefficient; *r*: relatedness; *Fst*: Wright's measure of population subdivision; *I*: Nei index; *E*: Euclidean distance. Statistical comparisons between ecological zones were conducted using non-parametric, Mann-Whitney tests (different letters indicate significant differences).

**Figure 4:**
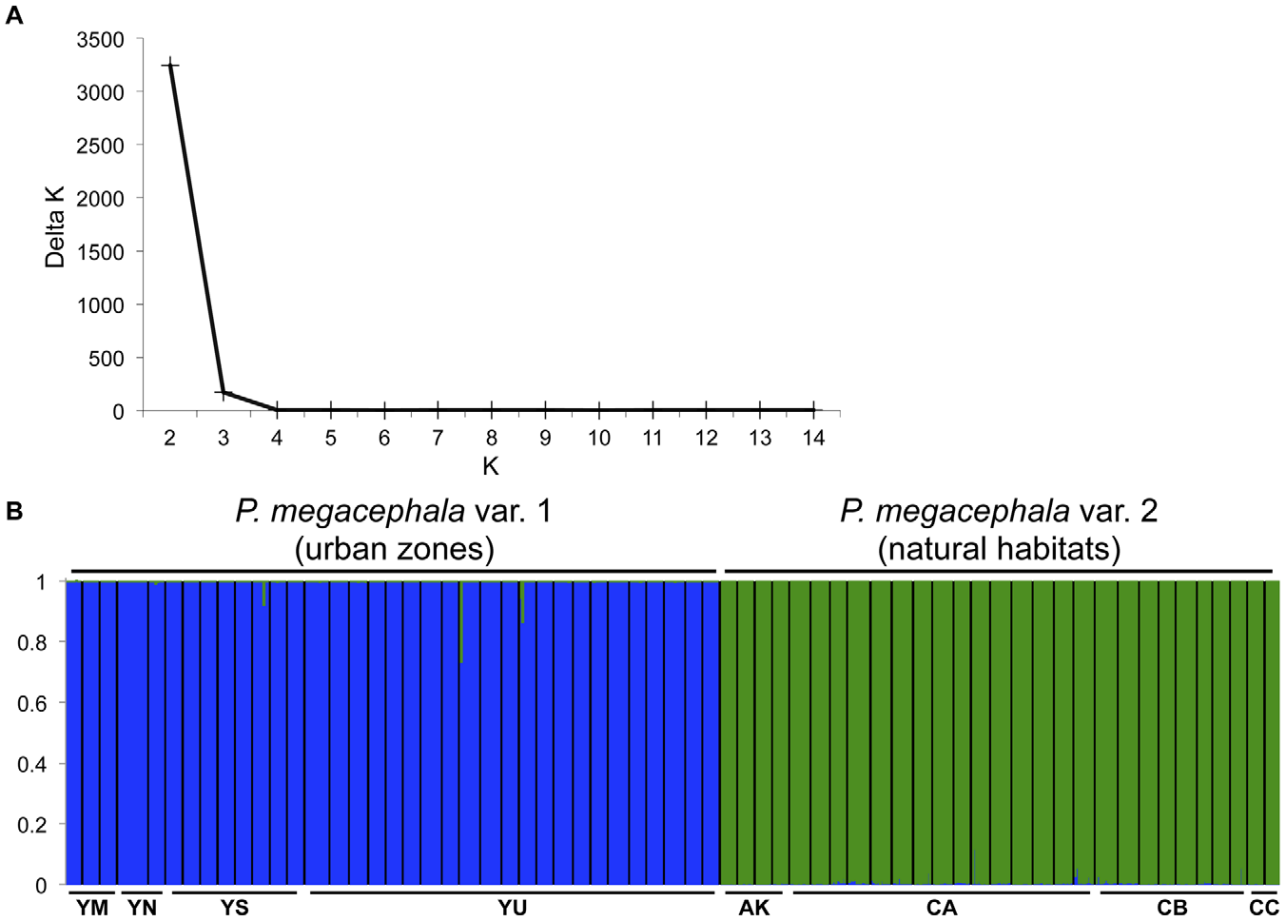
Bayesian cluster analysis. A: *ΔK* (a measurement of the rate of change in the structure likelihood function) values as a function of *K*, the number of putative supercolonies. In this case, *K* = 2. B: Graphical representation of the data set for the most likely *K* = 2, where each colour corresponds to a suggested cluster and each individual is represented by a vertical bar. The populations are indicated in the *X*-axis (AK: Akok; CA, CB and CC: Campo Forest Reserve; YM, YN, YS and YU: Yaoundé and its periphery). The *Y*-axis represents the probability for which an individual will be assigned to each cluster.

**Figure 5:**
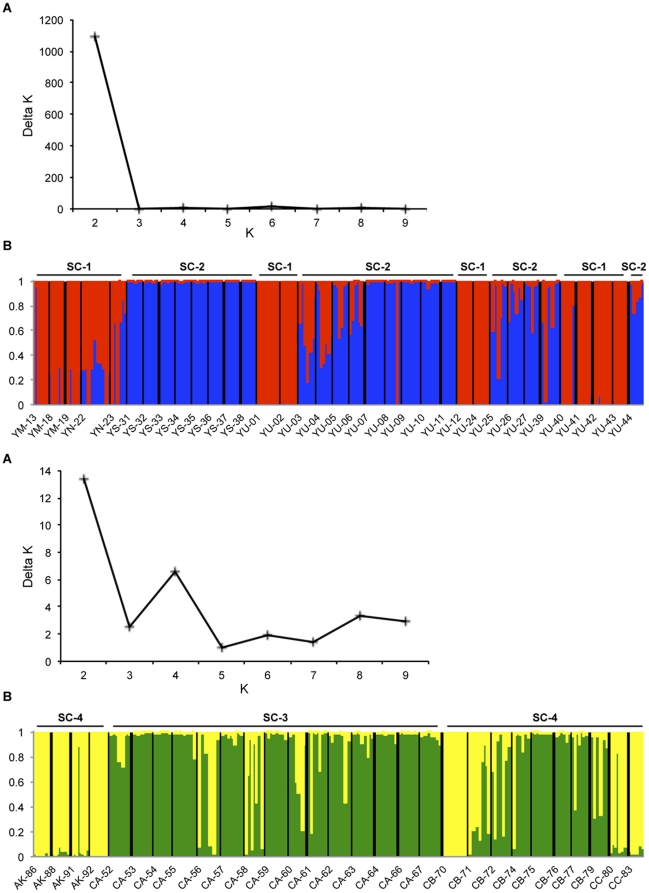
Inference of the number of genetic clusters (*K*) in populations of *P. megacephala* var. 1 (*i.e.* urban) (top) and *P. megacephala* var. 2 (*i.e.* rainforest) (bottom). A. *ΔK* (the standardized second order rate of change of ln P(*X*|*K*)) is plotted as a function of *K*. B. Proportional membership of *P. megacephala* workers to genetic clusters (*K*) for *K* = 2. Each vertical bar represents an individual whose estimated proportion of membership to either cluster (Y-axis) is indicated by the two different shades. Black lines separate individuals from different nests. Colours correspond to genetic clusters.

The mean genetic relatedness between workers from the same nest was significantly greater than zero for all of the populations studied (mean ± se = 0.680±0.029 and 0.699±0.022 for *P. megacephala* var. 1 and *P. megacephala* var. 2, respectively; Table 1). Similarly, workers within supercolonies were closely related (Table 2). In contrast, relatedness between pairs of supercolonies was low and not different from zero (mean ± se = 0.068±0.044, 95% confidence interval [−0.036;0.172] for *P. megacephala* var. 1 and 0.181±0.097 [−0.047;0.409] for *P. megacephala* var. 2). Accordingly, genetic differentiation estimated by the *Fst* index confirmed significant levels of population structure between supercolonies of a same phylogenetic group (*Fst* = 0.183 and 0.030 for *P. megacephala* var. 1 and *P. megacephala* var. 2, respectively) and low gene flows between supercolonies belonging to different phylogenetic groups (*Fst* varied from 0.216 (*Nm* = 0.91) to 0.319 (*Nm* = 0.53)). In line with these results, Bayesian cluster analyses of the microsatellite data set detected genetic substructures even without including the sampling locations of the individuals. The resulting posterior probabilities were highly concordant between the replicated runs, with the highest average value indicating the most likely number of population groups, *K*. Our whole data yielded a best estimate of *K* = 2 (Figure 4A), which was also confirmed by a peak in the *ΔK* statistic. Clusters C1 and C2 consisted of 35 and 29 nests, and corresponded exactly to the two cryptic species (Figure 4B). The probability for each worker to be correctly assigned to the phylogenetic group from which it was sampled was high. In addition, the Bayesian analyses applied to each cryptic species clearly indicated the presence of genetic clusters corresponding to the four supercolonies, as revealed through the behavioural assays (Figure 5).

**Table 2:** Statistics (mean ± se and [95% confidence interval]) for genetic and chemical analyses of the four supercolonies defined from behavioural assays.

	Genetic data						Chemical data	
	*A*	*Ar*	*H*	*f*	*r*	*Fst*	*I*	*E*
*P. megacephala* var. 1								
(urban populations)								
SC-1 (13 nest)	11.250±1.971	11.086±1.931	0.769±0.034	0.363±0.065	0.709±0.054	0.273±0.014	0.946±0.001	0.100±0.007
				[0.210–0.517]	[0.599–0.819]	[0.246–0.300]		[0.087±0.113]
SC-2 (22 nests)	8.250±1.485	7.880±1.455	0.596±0.071	0.207±0.078	0.663±0.036	0.146±0.005	0.926±0.013	0.172±0.010
				[0.022–0.392]	[0.587–0.739]	[0.135–0.157]		[0.153–0.192]
*P. megacephala* var. 2								
(rainforest populations)								
SC-3 (15 nest)	6.000±0.866	5.720±0.791	0.599±0.054	0.431±0.104	0.721±0.026	0.216±0.006	0.705±0.019	0.364±0.017
				[0.185–0.678]	[0.665–0.776]	[0.203–0.227]		[0.330–0.398]
SC-4 (14 nests)	5.625±0.754	5.499±0.731	0.606±0.047	0.254±0.099	0.676±0.036	0.242±0.012	0.801±0.018	0.455±0.008
				[0.019–0.489]	[0.598–0.754]	[0.219–0.265]		[0.439–0.471]

SC-1: nests YM-13, YM-18, YM-19, YN-22, YN-23, YU-01, YU-02, YU-12, YU-24, YU-40, YU-41, YU-42, YU-43.

SC-2: nests YS-31, YS-32, YS-33, YS-34, YS-35, YS-36, YS-37, YS-38, YU-03, YU-04, YU-05, YU-06, YU-07, YU-08, YU-09, YU-10, YU-11, YU-25, YU-26, YU-27, YU-39, YU-44.

SC-3: nests CA-52, CA-53, CA-54, CA-55, CA-56, CA-57, CA-58, CA-59, CA-60, CA-61, CA-62, CA-63, CA-64, CA-66, CA-67.

SC-4: nests AK-86, AK-88, AK-91, AK-92, CB-70, CB-71, CB-72, CB-74, CB-75, CB-76, CB-77, CB-79, CC-80, CC-83.

*A*: number of alleles; *Ar*: allelic richness; *H*: gene diversity; *f*: inbreeding coefficient; *Fst*: Wright's measure of population subdivision; *I*: Nei index; *E*: Euclidean distance.

Within supercolonies, differentiation between nests collected from Yaoundé and its periphery (*i.e. P. megacephala* var. 1) was statistically lower than differentiation between nests collected from the Campo Forest Reserve (*i.e. P. megacephala* var. 2) (*Fst* = 0.178±0.006 and 0.228±0.006, respectively; Mann-Whitney test, *p*<0.001). The maximum distance between nests in the same supercolony varied from 1 to 49 km; on the other hand, the minimum distance between nests belonging to two different supercolonies was 46 m (Figure 1).

Estimates of genetic diversity, inbreeding and relatedness coefficients within supercolonies are given in Table 2. Across the two cryptic species, supercolonies showed a positive and statistically significant *f* value, indicating non-random mating. Inbreeding coefficients ranged from 0.207 to 0.431, and corresponded to approximately 50 to 75% full-sib mating given the relationship *f* = *α*/(4-3*α*) where *α* is the frequency of sib-mating [42]. This is corroborated by the high average relatedness (± standard errors) between the queens and their mates (inferred from the sperm stored in queen's spermatheca) (0.694±0.038; 95% CI: 0.612–0.777). The mean genetic relatedness between cohabiting queens was high and equal to 0.634±0.075 (95% CI: 0.467–0.806). Estimates of the effective number of queens per supercolony – corrected for the effect of inbreeding - revealed a low degree of polygyny and corresponds to groups with 1–4 singly-mated queens. This is largely below the number of queens observed in the field (up to 71 queens were collected from a single nest of the population YS).

### Diversity and variation in cuticular hydrocarbon profiles (CHC)

Cuticular lipids yielded 47 peaks ranging in size from C19 to C40. Electronic and chemical ionizations did not show qualitative nor quantitative differences of CHC profiles isolated from urban populations (*P. megacephala* var. 1) with those previously described for Australian populations [34] (Nei's index of diversity, *P. megacephala* var. 1: mean ± se = 0.933±0.009; Australia: 0.938±0.002; Mann-Whitney *U*-test, *p* = 0.260). Conversely, diversity of profiles isolated from individuals collected in rainforest (*P. megacephala* var. 2) was significantly lower than that observed in Australian populations (*I* = 0.765±0.015; Mann-Whitney test, *p*<0.001). Ionisation mass spectrometry revealed the presence of lipids including linear alkanes, methyl-branched alkanes and alkenes [34].

**Figure 6:**
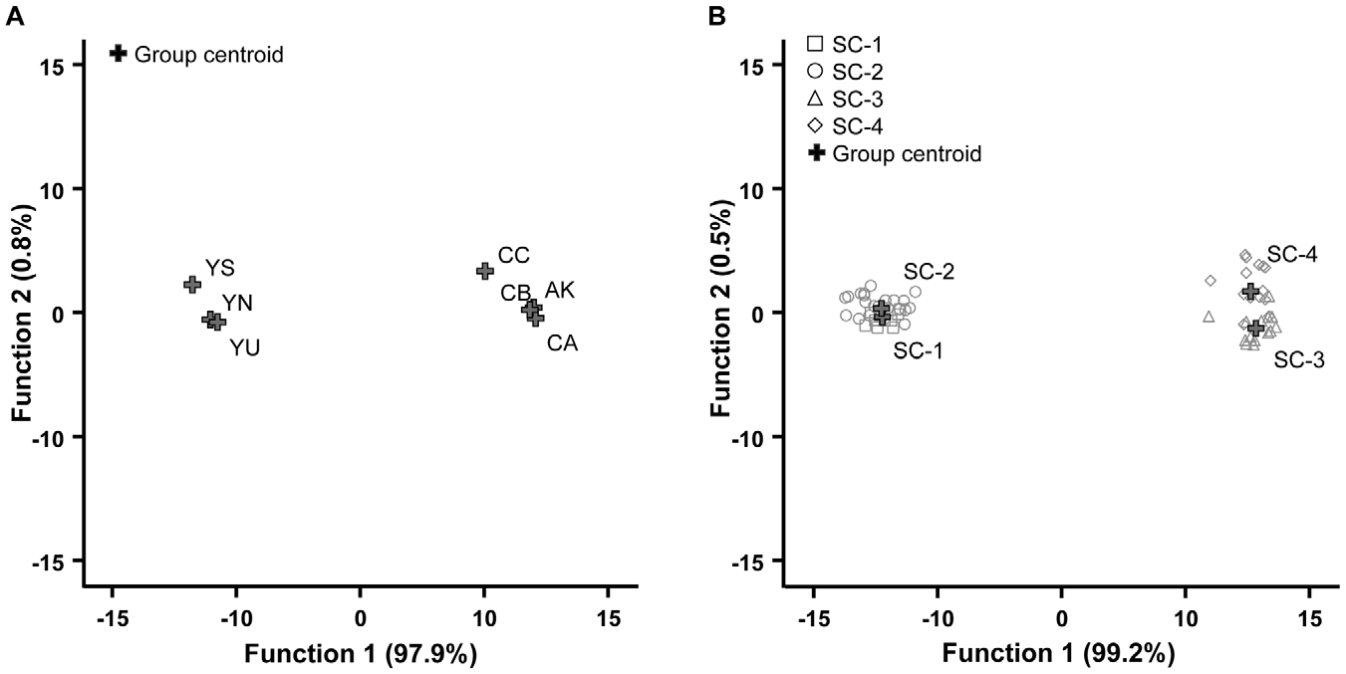
Chemical differentiation between the seven populations (A) and the four supercolonies (B). The differentiation is illustrated by a factor map of the two first axes of the canonical discriminant analysis on the relative proportions of cuticular lipids. Functions one and two account for 98.7% and 99.7% of the total variability among populations and supercolonies, respectively. Only group centroids are plotted on figure A. *P. megacephala* var. 1: populations YM, YN, YS, YU and supercolonies SC-1, SC2; *P. megacephala* var. 2: populations AK, CA, CB, CC and supercolonies SC-3, SC-4.

Discriminant analyses on 12 principal components (cross-validated) allowed 71.7% of the Cameroonian nests to be accurately classified in their phylogenetic group (YU 95.5%; YN 50.0%; YS 75.0%; CA 85.7%; CB 37.5%; CC 0%; AK 0%) (Figure 6A). The first and second canonical discriminant functions (CDFs) accounted for 97.9% and 0.8% of the total variance, respectively. Canonical correlation values (range: 0.379 to 0.997) indicated that the canonical varieties might explain the differences between nests from the two cryptic species. Likewise, the cuticular hydrocarbon profiles of nests clustered according to behavioural and genetic supercolonies (Figure 6B). The first two principal components accounted for 99.7% of the overall variance between groups; the canonical correlation values associated with these two CDFs were, respectively, Cc1 = 0.996 and Cc2 = 0.626. Overall, 70.0% of the cross-validated samples were correctly assigned to their supercolonies by the two CDFs (SC-1 60.0%; SC-2 72.7%; SC-3 85.7%; SC-4 57.1%).

Within each cryptic species, the diversity of the chemical profiles measured by the Nei index *I* did not differ between nests, nor between supercolonies (Kruskal-Wallis tests followed by Dunn's procedures, all *p*>0.05; Table 1). In contrast, profiles of *minor* collected from the rainforest were significantly less diverse than the profiles of urban populations (*I* = 0.765±0.015 and 0.933±0.009, respectively; Mann-Whitney test, *p*<0.001). The similarity in chemical profiles estimated by the Euclidean distances *E* was different between the two ecological zones. The chemical differentiation between nests was lower in *P. megacephala* var. 1 (urban) than in *P. megacephala* var. 2 (rainforest) populations (*E* = 0.151±0.006 and 0.444±0.006, respectively; Mann-Whitney test, *p*<0.001; Table 1).

### Associations between behavioural, genetic, chemical and spatial data

In populations of *P. megacephala* var. 1, correlation analyses showed that aggressiveness was closely related to genetic (Mantel test, *r* = 0.487, *p*<0.001) and spatial distances (*r* = 0.502, *p*<0.001). In contrast, chemical distances had no influence on aggressiveness (*r* = −0.054, *p* = 0.597). In *P. megacephala* var. 2, aggressiveness was not associated with any of the three distances estimated (all *p*>0.527). Genetic differentiation, CHC profile dissimilarity and spatial distances were all positively correlated (all *r*>0.276, *p*<0.001), except in *P. megacephala* var. 2 where chemical and spatial distances were not associated (*r* = 0.036, *p* = 0.430). For both cryptic species, the diversity of cuticular compounds measured by the Nei index *I* was associated with neither the allelic richness (Spearman's rank correlations, *p*>0.569), nor with the genetic diversity (*p*>0.366).

### Contributions of various levels of population structure to patterns of chemical and genetic variation

In the analyses of molecular variance (AMOVA), the total genetic differentiation among populations was 0.482 (*F_ST_*) of which 0.352 (*F_RT_*) was due to the among-cryptic species component and 0.200 (*F_SR_*) was due to the among-population within-cryptic species component (Table 3). AMOVA analyses of chemical compound differentiation provided similar results, with differentiation among populations being 0.412 of which 0.367 was due to the among-species component and 0.071 to the among-population within-species component (Table 3). The distributions of genetic and chemical variations were comparable whether we used populations or supercolonies as reconstructed by the behavioural assays (Table 4).

**Table 3:** Results of the hierarchical analyses of molecular variance (AMOVAs) evaluating the amount of genetic and chemical variance between and within groups.

Source	*df*	MS	Percentage of variation (%)	*F* value	*p*
Genetic variance					
Between cryptic species (*F_RT_*)	1	2.849	35%	0.352	0.001
Between populations (*F_SR_*)	6	0.282	13%	0.200	0.001
Within populations (*F_ST_*)	56	0.110	52%	0.482	0.001
Total	63		100%		
Chemical variance					
Between cryptic species (*F_RT_*)	1	3.759	37%	0.367	0.001
Between populations (*F_SR_*)	5	0.256	5%	0.071	0.021
Within populations (*F_ST_*)	54	0.152	59%	0.412	0.001
Total	60		100%		

Nests were assigned to their population.

**Table 4:** Results of the hierarchical analyses of molecular variance (AMOVAs) evaluating the amount of genetic and chemical variance between and within supercolonies.

Source	*df*	MS	Percentage of variation (%)	*F* value	*p*
Genetic variance					
Between cryptic species (*F_RT_*)	1	2.849	30%	0.296	0.001
Between populations (*F_SR_*)	2	0.805	21%	0.304	0.001
Within populations (*F_ST_*)	60	0.104	49%	0.510	0.001
Total	63		100%		
Chemical variance					
Between cryptic species (*F_RT_*)	1	3.759	36%	0.356	0.001
Between populations (*F_SR_*)	2	0.382	5%	0.082	0.006
Within populations (*F_ST_*)	57	0.153	59%	0.409	0.001
Total	60		100%		

## Discussion

Our results show that two cryptic species of *Pheidole megacephala* cohabit in Cameroon, and that these species differ in their ecological niche: *P. megacephala* var. 1 occupies urban areas and *P. megacephala* var. 2 is found in rainforests. These two reproductively isolated groups may have cohabited for a long time in Cameroon. Alternatively, urban populations may result from a recent introduction from other populations originating from Africa, or not. The presence of private alleles supports a reduced gene flow between species. Despite this reproductive isolation, our data show that they share similar reproductive and dispersal strategies. Within each phylogenetic group, nests are organized into mutually aggressive supercolonies, and supercolonies are genetically differentiated. Genetic differentiation between supercolonies may result from at least two factors. First, new queens are recruited among daughters, as revealed by the high relatedness among cohabiting queens (*r* = 0.637±0.075). Second, mating takes place in the nests between related individuals (*r* = 0.694±0.038 between a queen and her mate), and results in a relatively high level of inbreeding. This reproductive pattern and the significant correlation between the genetic and geographical distances indicate that the main mode of colony reproduction occurs *via* dependent foundation, whereby a queen and a few workers leave the natal nests to start a new colony nearby. These breeding and dispersal strategies contribute maintaining genetic homogeneity in each supercolony, and simultaneously generate high levels of inbreeding. In Hymenoptera, a direct cost of inbreeding is the production of homozygous diploid and sterile males. However, in spite of the high inbreeding coefficient, no such diploid males were found in our sample (*N* = 88, unpublished data). As suggested for other Hymenoptera [34], [43], the male-haploid sex determining system may help purging deleterious alleles [44], [45].

The two cryptic species show life history traits that are characteristic of introduced populations, both in Yaoundé where preliminary field observations indicate that *P. megacephala* is dominant in the human-disturbed zone and could negatively affect native ant fauna [personal observations, Mbenoun et al. unpublished data] and [36], and in less disturbed habitats of the Campo Forest Reserve. Nests are numerous and form supercolonies extending over 1 to 49 km (Figure 1), with high numbers of reproductive queens. In Yaoundé and its periphery, the supercolonies are very close to each other, with nests belonging to different supercolonies being separated by only 46 m. Interestingly, field observations showed that the contact zone is propitious to the presence of two other ant species, namely *Odontomachus troglodytes* and *Myrmicaria opaciventris*. In contrast to Australian populations, *P. megacephala* in Cameroon does not form a huge, single unicolonial population spreading over thousands of kilometres, but a mosaic of mutually aggressive supercolonies. Such a difference between populations has been documented for two other invasive ants between invaded and native ranges. Supercolonies of the Argentine ant *Linepithema humile* have been found spanning 6000 kilometres across Europe [25] and extending for 1000 km in California [24], whereas populations are organized into smaller supercolonies in its native range [23], [46], [47]. Likewise, a single 450-km-long supercolony of the little fire ant *Wasmannia auropunctata* has been reported in New Caledonia [48], while supercolonies encountered in the native range are several orders of magnitude smaller [28], [49].

**Figure 7:**
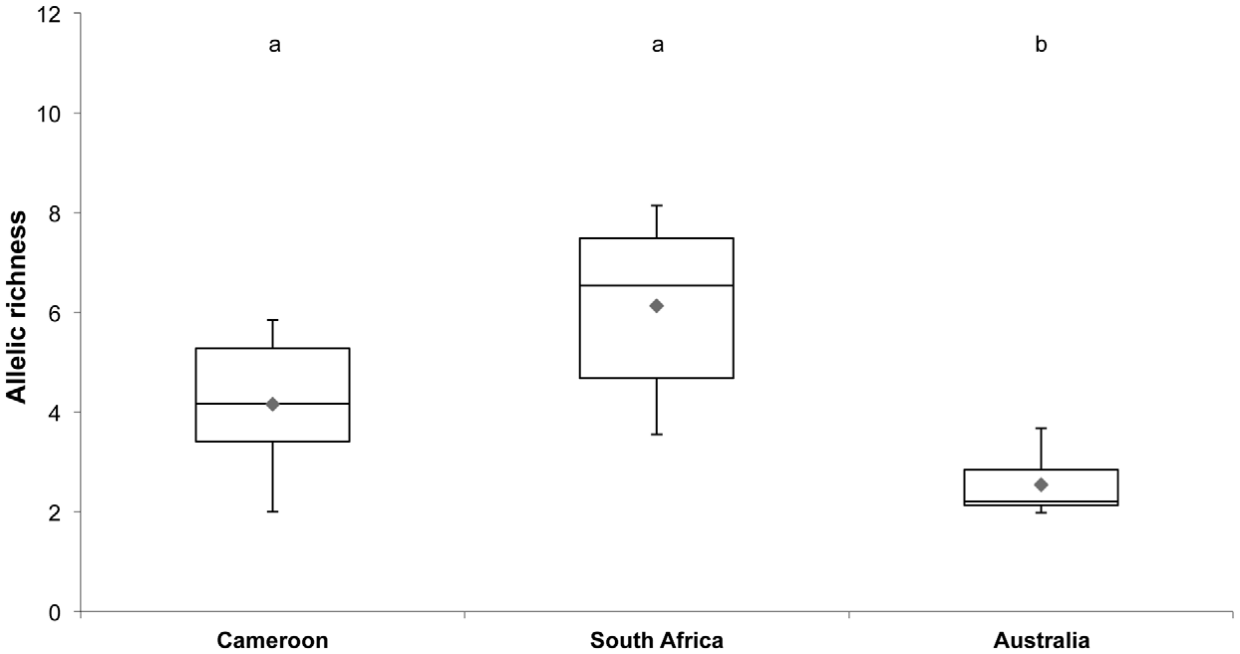
Box-plot of the allelic richness estimated at eight microsatellites loci for samples collected from Cameroon (*P. megacephala* var. 1), South Africa [**40**] and Australia [**34**]. Lower case letters link groups that are not statistically distinguishable using post-hoc tests (Tukey's HSD) at *α* = 0.05.

Our data also reveal that populations collected in urban areas in Cameroon belong to the same phylogenetic group than those from Australia and South Africa. However, genetic diversity and allelic richness appear significantly different among Cameroon (*P. megacephala* var. 1 populations; present study), South Africa [40] and Australia [34] (one-way ANOVAs followed by Tukey's post-hoc tests, *F*>14.151, *p*<0.001; Figure 7). Australian populations exhibit a remarkably low genetic diversity compared to South African and Cameroonian populations; in contrast, no difference occurs between the African populations (Figure 7). An analysis of molecular variance (AMOVA) shows that of the total molecular variance, 34% (*F_RT_* = 0.340) is attributed to divergence among countries, 9% (*F_SR_* = 0.135) to populational differences within region, and 57% (*F_ST_* = 0.429) to individual differences within populations (Table 5). Such genetic changes have been studied in detail in the Argentine ant *L. humile* [24], [25], the fire ant *Solenopsis invicta* [50] and the little fire ant *W. auropunctata* [51], and have been shown to result from bottlenecks at introduction. Reduced genetic diversity may have negative effects by limiting population growth [52], [53] and by diminishing the ability to adapt to a new environment [54]. However, successive introductions with interbreeding between introduced populations [55], [56], [57], and the purge of deleterious alleles linked to the drastic reduction in genetic diversity [58], [59], [60] may greatly limit the negative effects of low genetic diversity, as exemplified by the invasive success of introduced populations. According to a stepping-stone model, once populations are established in urban areas, they probably serve as sources for secondary introductions elsewhere, and as one goes along introductions, nests form networks. Thus, supercolonies can be considered as a step along a continuum of social organization where multicoloniality and unicoloniality represent the two extremes. This process was documented for the Argentine ant *Linepithema humile* [61], [62]: its extent from Rosario (Argentina) to California (US) *via* Buenos Aires and the south-eastern United States was accompanied by an increase in the size of the colonies. Phylogenetic relationships and genetic differentiation between supercolonies, as well as their allelic composition raise the possibility that urban *P. megacephala* colonies could be the source of introduced populations.

**Table 5:** Hierarchical analysis of molecular variance (AMOVA) with country as grouping factor (Cameroon, *n* = 304; Australia, *n* = 419; South Africa, *n* = 20).

Source	*df*	MS	Percentage of variation (%)	*F* value	*p*
Between countries (*F_RT_*)	2	526.586	34%	0.340	0.010
Among populations (*F_SR_*)	6	54.041	9%	0.135	0.010
Within populations (*F_ST_*)	1477	2.095	57%	0.429	0.010
Total	1485		100%		

Cuticular lipids present on the surface of the cuticle of ants mediate nestmate recognition between individuals [20], [21], [22]. Ants react according to either a gradual or a threshold effect [63]. In the first situation, they exhibit scaled aggressiveness from rejection to acceptance, the intensity of the response depending on the qualitative and/or quantitative variations between the cuticular profiles of the protagonists [21], [22], [63]; in the second situation, ants are accepted or rejected. Our data do not show a correlation between aggressiveness and the differentiation of the cuticular hydrocarbon profile; rather, they suggest that the behaviour of *P. megacephala* workers depends on a threshold beyond which the response is aggressive. This threshold could be expressed through variations in the relative abundance of chemical compounds in the cuticular profile, or through the existence of chemical compounds specific to the supercolony. In the yellow crazy ant *Anoplolepis gracilipes*, aggressiveness between supercolonies increases with the dissimilarity in cuticular hydrocarbon profiles, and the proportion of qualitatively different cuticular hydrocarbon profiles is positively correlated with the proportion of alleles that differed between supercolony pairs [64]. However, the composition of the cuticular profiles is generally determined by the interaction between heritable and environmental cues derived from food or nesting materials. Our data show that cuticular profiles in *P. megacephala* do not differ between supercolonies belonging to the same environment, despite a significant genetic differentiation between supercolonies. In addition, our results indicate that there is no association between genetic and chemical diversity. This suggests that the presence of private alleles is not expressed in the cuticular compounds specific to the supercolony. Therefore, it seems likely that the environment plays a significant role in the cuticular cues involved in nestmate recognition in this species. However, we cannot excluded that nestmate discrimination in *P. megacephala* relies upon highly sensitive responses to few specific compounds masked in the overall odour profile and not detected in our analyses [65], and/or that only some classes of chemicals or individual compounds involved in nestmate recognition are correlated with genetic and behavioural patterns [66], or that this species uses chemicals other than cuticular hydrocarbons for nestmate recognition [67]. Further investigations consisting in treating individuals with known compounds, or to transfer cuticular lipid extracts between ants from different supercolonies [68] should be conducted to clarify the support of nestmate recognition in *P. megacephala*. Furthermore, our data reveal a greater chemical diversity in cuticular profiles for ants collected from urban areas than for those collected from the Campo Forest Reserve habitats. Our sampling was conducted during the dry season, which is less favourable to population and colony growth. At this period of the year, it seems likely that urban areas offer a greater diversity in the choice of habitats and food sources than forest areas. Consistent with this, we observed that ants in Yaoundé were opportunistic generalists and used a variety of nesting sites and food sources, while in the forest they were usually confined to plant roots (*e.g. Pennisetum purpureum* Schumacher (*Poaceae*) and *Costus afer* Ker-Gawler (*Costaceae*)) where they tended aphids. Chemical differentiation between urban and rainforest populations may therefore be induced by environmental conditions, but it may also stem from phylogenetic differences. Whether these chemical divergences lead to or result from reproductive isolation could remain crucial for understanding the evolutionary history of the two cryptic species.

Our study shows that the African big-headed ant *P. megacephala* forms unicolonial populations in Cameroon, both in natural habitats and urban areas. It has been suggested that unicoloniality is a key factor responsible for the ecological dominance of ants. Many invasive ant species (*e.g. Linepithema humile* [69], *Anoplolepis gracilipes* [70], *Wasmannia auropunctata* [71], *Solenopsis invicta* [72], *Lasius neglectus* [73], *Paratrechina longicornis* [74], *P. megacephala* [34] and *Tapinoma sessile* [75]) are indeed unicolonial. However, recent findings challenge the role of unicoloniality in the invasive success of introduced populations and have highlighted the evolutionary contradictions posed by this social organization in terms of kin selection. First, introduced populations of a given species do not necessarily have a different social or genetic structure than native ones [23], [28], [47], and native populations may also attain high densities [76], [77]. Second, unicolonial populations are characterized by the presence of numerous egg-laying queens, so that workers display reproductive altruism towards random members instead of relatives [78], [79]. Interestingly, our data show that this is not the case in the present study, where relatedness between workers within supercolonies is high (*r* = 0.692±0.014) due notably to a high kinship among breeders. Our study emphasizes a fundamental distinction between African and introduced populations in Australia in terms of nest structure. In Australia, populations are organized into a single supercolony spreading over 3000 km [34]. In Cameroon, on the other hand, *P. megacephala* forms a mosaic of mutually unrelated supercolonies within which individuals are related. In this context, competition (*e.g.* conquering new territories or food sources) between supercolonies favours reproductive altruism within supercolonies [23], [80], [81], and hinders the propagation of selfish detrimental traits by selectively eliminating less competitive supercolonies to the advantage of more competitive ones [78], [82].

One commonly proposed explanation for invasive success is the enemy release hypothesis (ERH), which states that the lack of natural predators and competitors in an exotic environment favours the successful establishment and spread of species outside of their native range [83]. Our study shows that *P. megacephala* var. 1 share behavioural and evolutionary traits that are characteristic of introduced of *P. megacephala* populations in Australia. Moreover, field observations in Cameroon indicate that this species largely dominates other native ants in urban habitats. The ERH may act in synergy with other factors facilitating the introduction and naturalization of *P. megacephala*. First, propagules composed of a queen and a few workers may experience high survivorship and rapid growth. A study conducted in Australia showed that the invasion by *P. megacephala* is consistent with the introduction of only six haploid genomes [34]. Second, *P. megacephala* combines a high aggressiveness towards other ant species with highly efficient predatory capacities. In invaded ranges, this species captures many different types of prey and is more efficient than native ants in preying on termites [37]. This stems from the effective exploitation of the landmarks deposited by termites and competing ants, and the efficient short- and long-range recruitment of conspecifics. In the ant *Wasmannia auropunctata*, one of the most invasive and destructive pests in the world [84], the combination of several life history traits seems particularly relevant in explaining its ecological dominance. (*i*) Its clonal reproductive system, whereby males and females are clonally produced [85], maintains favourable combinations of genes over time and thus offers an adaptive advantage over populations that reproduce sexually. (*ii*) Its unicolonial population structure provides numerical and competitive advantages over multicolonial ants [48]. (*iii*) Its proximity to humans allows it access to profoundly modified habitats that may, over time, increase its ability to become invasive [84], [86]. In fact, the main factor associated with the ecological dominance of *W. auropunctata* is the human activities [49]; its reproductive system and social organisation strengthen the chances of successful invasion, but are neither necessary nor enough to explain the success of the invasion. Likewise, the success of other invasive ant species such as *Solenopsis invicta* [87], *Anoplolepis gracilipes* [88], *Linepithema humile* [89], *Paratrechina longicornis* [90] and *Lasius neglectus* [91] also seems correlated to anthropogenic disturbances. Biotic homogenization and habitat degradation within urbanized regions can certainly promote the evolution and the expression of invasive characteristics [92].

In conclusion, our study shows that Cameroon hosts two cryptic, reproductively isolated species of the big-headed ant *P. megacephala*. These species are restricted to certain ecological zones: *P. megacephala* var. 1 is found in urban areas and *P. megacephala* var. 2 in rainforests. Urban populations are phylogenetically close to populations in Australia, Mauritius, South Africa and Madagascar. Rainforest populations have high homologies with populations in Gabon, Madagascar and Comoros. Populations of both cryptic species are organised into a mosaic of supercolonies and adopt a unicolonial structure. Further analyses should decipher the cryptic species complex of *P. megacephala* and to identify the sub-species (if not all) particularly suited to adapt to and invade new environments. The ecological and evolutionary strategies adopted in native areas should be considered as a first phase in the expansion of and invasion by this species. More attention should also be placed on how anthropogenic activities affect the biotic and abiotic conditions of the environment, and to what extent invasional meltdown processes [93] could facilitate invasion. The next step in understanding the worldwide expansion of the ant *P. megacephala* will be to investigate the routes and means of introduction to prevent new invasions.

## Materials and Methods

### Field collection and sampling

Sixty-four nests (*i.e.* an aggregation of workers, brood, and/or queens in a single place) were sampled from eight populations in Cameroon in February 2009 (Figure 1 and Table 1; The Ministry of Scientific Research and Innovation provided the research permit # 019/MINRESI/B00/C00/C10/C13). Nests were collected from two ecological zones, a rainforest (29 nests sampled from along forest tracks in the Campo Forest Reserve) and a human-disturbed area (35 nests located in Yaoundé and its periphery). The Campo rainforest belongs to the Biafran Atlantic low altitude forest district; it is characterized by a rich and diverse flora dominated by the plant family *Caesalpiniaceae* [94], [95]. The nests were located mainly below the roots of plants such as *Pennisetum purpureum* Schumacher (*Poaceae*) and *Costus afer* Ker-Gawler (*Costaceae*), or, more rarely, in dead branches or termite mounds. Different species of the genus *Pheidole* and species belonging to other genera (*e.g. Camponotus*, *Tetramorium*, *Pachycondyla*, *Myrmicaria*, *Cataulacus*, *Dorylus*, *Odontomachus*, *Oecophylla*) were sympatric with *P. megacephala*. In contrast, the vegetation in the Yaoundé urban area belongs to the semi-deciduous forest type, but currently is a mixture of forest relics on hill summits and garden crops (urban agriculture) along river and inland valleys. In this zone, *Pheidole megacephala* is opportunist; it nests under stones or dead branches, and is found in gardens, kitchen-gardens and dwellings. Only a few other species lived in the proximity of the *P. megacephala* nests, namely *Odontomachus troglodytes*, *Monomorium bicolor*, *Crematogaster clariventris*, *Lepisiota* sp. and *Myrmicaria opaciventris*. Samples of workers and, when available, queens and males were collected from each nest and used for the behavioural assays. *Pheidole megacephala* has a pronounced worker caste polymorphism: major workers are considerably larger than *minor* and have disproportionately large heads. The *minor* are more active than the *major*, notably in exploring the ground and in alerting nestmates of the presence of intruders (as shown in *P. guilelmimuelleri* and *P. pubiventris* [96], *P. morrisi* [97] and *P. pallidula* [98]). A sub-sample of 50 *minor* from each nest was used for chemical extraction, and then stored in ethanol for subsequent genetic analyses.

### Morphological studies

Morphological characteristics of specimens were assessed using a stereomicroscope and a scanning electron microscope. The maximum head width (across compound eyes) of 12 minors per population was determined at the nearest 0.005 mm by using a stereomicroscope (Wild M3, Wild Heerbrugg, Switzerland) at a magnification of 40×. Head width is an accurate estimate of size, commonly used as a dependent variable in studies of allometry [99], [100]. Repeated measures differed on average by only 0.03 mm and were highly correlated (*n* = 32, Pearson's correlation, *r_P_* = 0.803, *p* = 0.001). A scanning electron microscope (JSM-6480LV, JEOL; Tokyo, Japan) was used to study the shape and surface details of two *minor* and one *major* of each ecological zone. To this purpose, samples were dehydrated in ethanol, critical point air-dried and coated with gold using an ion sputter (JFC-1300 Fine Coater, JEOL).

### Behavioural assays

Behavioural assays were conducted by confronting minor workers. Intraspecific aggressiveness was quantified between pairs of *minor* according to previously described protocols [34], [101]. Standardized aggressiveness tests were conducted during 5 min. after the first interaction between two individuals placed together in a neutral arena (diameter = 2 cm, height = 1 cm, sides coated with Fluon™). Interactions were scored on a scale from 1 to 4: levels 1 (short antennations (<2 sec)) and 2 (prolonged antennations) were considered as non-aggressive behaviours, whereas levels 3 (lunging and attempts at biting) and 4 (prolonged biting and pulling) were considered as agonistic. Five trials, each involving different *minor*, were conducted for each pair of nests and the highest score was averaged across trials.

Interactions between individuals belonging to different nests, whether from the same or different sites, were considered as experimental assays. Interactions between individuals from the same nests were considered as control assays. In addition, we set up encounters between *major* from different nests to confirm that workers from both castes did not behave differently when confronted with non-nestmates.

### Diversity and variations in cuticular hydrocarbon profiles

Protocols for the extraction of cuticular hydrocarbons and gas-chromatography, mass-spectrometry (GC-MS) analyses are described elsewhere [34]. In short, samples of ant cuticular hydrocarbons were extracted from 50 *minor* per nest and placed in 1 ml of cyclohexane during 5 min; the cyclohexane was evaporated and the extract was re-diluted in 5 µl of cyclohexane. We injected 1 µl of the extract into a Thermo Polaris Q™ electron impact ion trap mass spectrometer interfaced to a Thermo Trace GC Ultra™ gas chromatograph (Thermo Finnigan; Austin, Texas, USA). Qualitative and quantitative data were obtained by running the Thermo Xcalibur™ data system (Thermo Finnigan; Austin, Texas, USA). Cuticular profiles obtained for three nests of the population YM did not present clear patterns, and were therefore discarded from the analyses. We used the relative amount (*i.e.* area) of the peaks corresponding to cuticular lipids as a quantitative measurement for each profile. Compounds with a relative area of less than 2% in all profiles were excluded from the data. Cuticular lipids were identified by analysing their mass spectra produced by both electron impact and chemical ionization with methane.

We estimated the chemical diversity and similarity of cuticular hydrocarbon profiles using the Nei index *I* and Euclidian distances *E*, respectively [34]. To visualise the differences in cuticular hydrocarbon profiles, we conducted a canonical discriminant function analysis based on the relative amounts of all identified compounds.

### Genetic analyses

DNA was isolated through phenol/chloroform extractions and ethanol precipitation by following standard protocols [102].

The phylogenetic relationships between collected samples were inferred from a 650 base-pair (bp) region near the 5′ terminus of the CO1 gene. COI sequences were generated from a subset of the individuals sampled (4 individuals per population) using barcoding protocols [39], [103]. Full-length COI barcodes (658 bps) were amplified using the standard insect primers LepF1/LepR1 (LepF1: 5′-ATTCAACCAATCATAAAGATATTGG-3′; LepR1: 5′-TAAACTTCTGGATGTCCAAAAAATCA-3′). PCRs were carried out in 25 µl reaction volumes containing 14 µl of distilled water, 5 µl of genomic DNA, 2.5 µl of Qiagen 10× buffer (providing a final concentration of 1.5 mM MgCl2), 2 µl 2.5 mM of dNTP, 0.625 µl 100 mM of forward and reverse primers and 0.250 µl of Qiagen Taq DNA polymerase. The PCR thermocycling profile consisted of one cycle of 2 min at 94°C, five cycles of 40 sec at 94°C, 40 sec at 45°C, and 1 min at 72°C, followed by 36 cycles of 40 sec at 94°C, 40 sec at 51°C, and 1 min at 72°C, with a final step of 5 min at 72°C. Products were visualized on a 2% agarose gel and samples containing clean single bands were sequenced using BigDye v3.1 on a 48-capillary 3730 DNA Analyzer (Applied Biosystems). The forward and reverse sequences for each sample were inspected and aligned using CodonCode Aligner 3.7.1.1 (CodonCode Corporation, Dedham, MA). Programs freely available from the phylogeny.fr web server [104] (Muscle for multiple alignment [105], Gblocks for alignment curation [106], PhyML for phylogeny [107] and TreeDyn for tree drawing [108]) were used to reconstruct a phylogenetic tree from our set of sequences. The sequences of *Pheidole sexspinosa* and *P. xerophila* were used as sister species and those of *Aphaenogaster senilis* as outgroup [38].

Genetic diversity and population genetic structure were investigated by genotyping eight statistically independent microsatellite loci [34]. The PCR amplification was carried out in 10 µL volumes. PCR products were separated on a 48-capillary 3730 DNA Analyzer (Applied Biosystems), and an internal size standard (GeneScan™ 350 ROX™, Applied Biosystems) was run in every sample. The lengths of the PCR products were determined using GeneMapper software (Applied Biosystems) and used to construct a multi-loci genotype for each individual.

Genetic diversity parameters (*i.e.* the number of alleles, allelic richness, expected and observed heterozygosities) and fixation indices were estimated using Fstat 2.9.3 [109] and averaged across loci. Regression relatedness and the jackknife estimates of the standard errors were calculated with the program Relatedness 5.08 [110], [111]. The mean genetic relatedness among *minor* was estimated by including all individuals as reference. The effect of inbreeding on relatedness was corrected by using the estimator r* [42]. The number of singly-mated, related and equally reproducing queens per nest was inferred from relatedness values among nestmate *minor* [112]. The assumptions of single mating and equal partitioning of reproduction among queens are corroborated by a previous study [34], and that nestmate queens are related is shown herein (see Results).

The most likely number of genetic clusters (*K*) reflecting the population genetic sub-structure of our data set was characterised with the Bayesian model-based clustering method implemented in Structure v2.3.2 [113]. For this purpose, the program was run without population information under the admixture model (individuals may have mixed ancestry) and independent allele frequencies. The length of the burn-in period was 10,000 and the number of Markov chain Monte Carlo replications after the burn-in was 10,000. Ten independent chains were run for each *K* from *K* = 2 to *K* = 64. The *ad hoc* statistic *ΔK* based on the rate of change in the log probability of data between successive *K* values was used to find the most likely value of *K* [114], [115].

### Associations between behavioural, genetic, chemical and spatial data

Correlations between the geographical origin, genetics, chemical composition and behaviour of *P. megacephala minor* were considered through Mantel correlation tests [116] based on 999 permutations and by using GenAlEx 6 [117]. The independent influences of the genetic, chemical and spatial distances on workers' aggressiveness were estimated through a stepwise multiple regression analysis [118]. If not stated otherwise, statistical tests were carried out with the computer program Spss Statistics 19 (Spss Inc., 1989–2010).

### Contributions of various levels of population structure to patterns of chemical and genetic variation

Hierarchical analyses of molecular variance (AMOVA) of the eight populations (*i.e.* collection sites) nesting within the two ecological zones (*i.e.* rainforest and human-modified habitats) were conducted to quantify the distribution of genetic and chemical variations between populations, zones and populations within zones. The significance of each variance component was tested using a random permutation test (999 permutations) using GenAlEx 6 [117].

## Supporting Information

Figure S1 ***Minor*** **of** ***P. megacephala*** **var. 1 (A) and** ***P. megacephala*** **var. 2 (B) in lateral views.** Magnification and scale bar are indicated for each scanning electron microscopic image.
(TIF)

Figure S2 **Lateral views of the propodeum showing shorter spines in** ***P. megacephala*** **var 1. than in** ***P. megacephala*** **var. 2 (B).** Magnification and scale bar are indicated for each scanning electron microscopic image.
(TIF)

Figure S3 **Details in lateral views of hairs present on the petiole.** Magnification and scale bar are indicated for each scanning electron microscopic image. The terminal part of the hairs forms a point in *P. megacephala* var. 1 (A) and a brush in *P. megacephala* var. 2 (B).
(TIF)
