# Supplementary figures and images for: Genetic Structure, Nestmate Recognition and Behaviour of Two Cryptic Species of the Invasive Big-Headed Ant Pheidole megacephala
Source: PLoS One. 2012 Feb 21;7(2):e31480. doi: 10.1371/journal.pone.0031480 (PMC3284284; doi:10.1371/journal.pone.0031480)

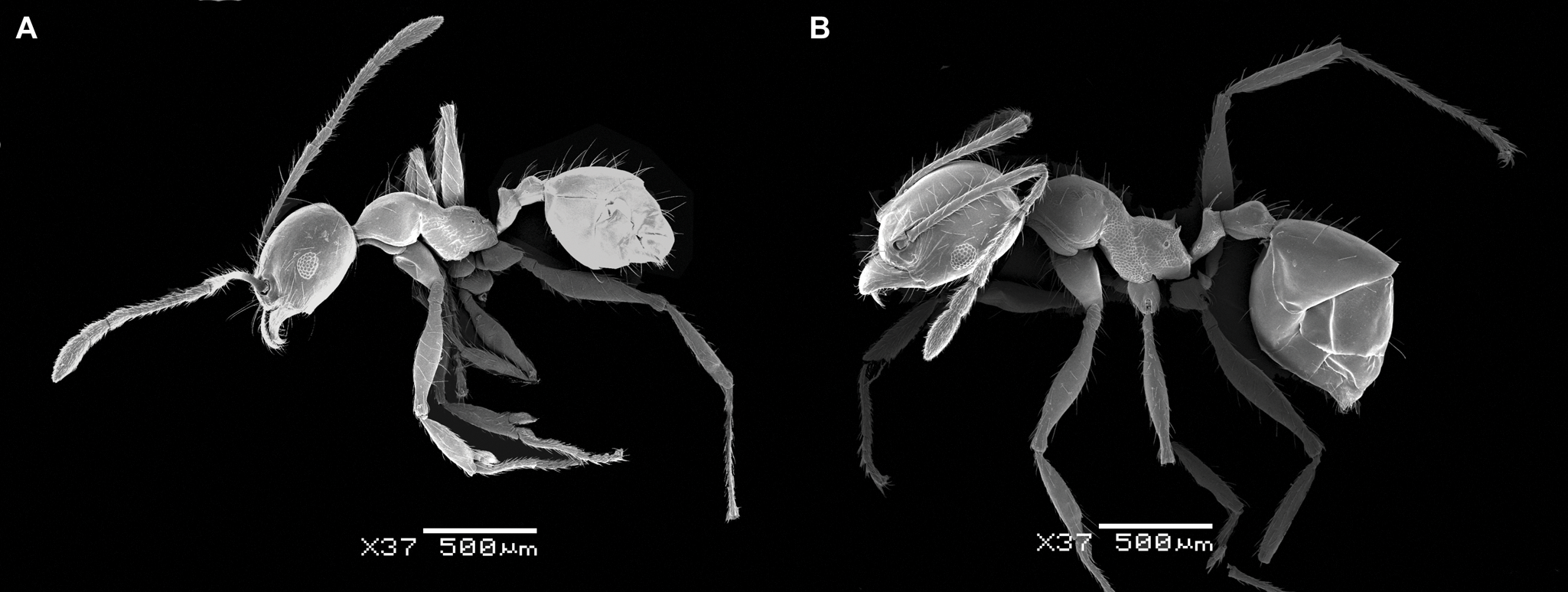

Supplement: Figure S1 — Minor of P. megacephala var. 1 (A) and P. megacephala var. 2 (B) in lateral views. Magnification and scale bar are indicated for each scanning electron microscopic image. (TIF) [file pone.0031480.s001.tif]

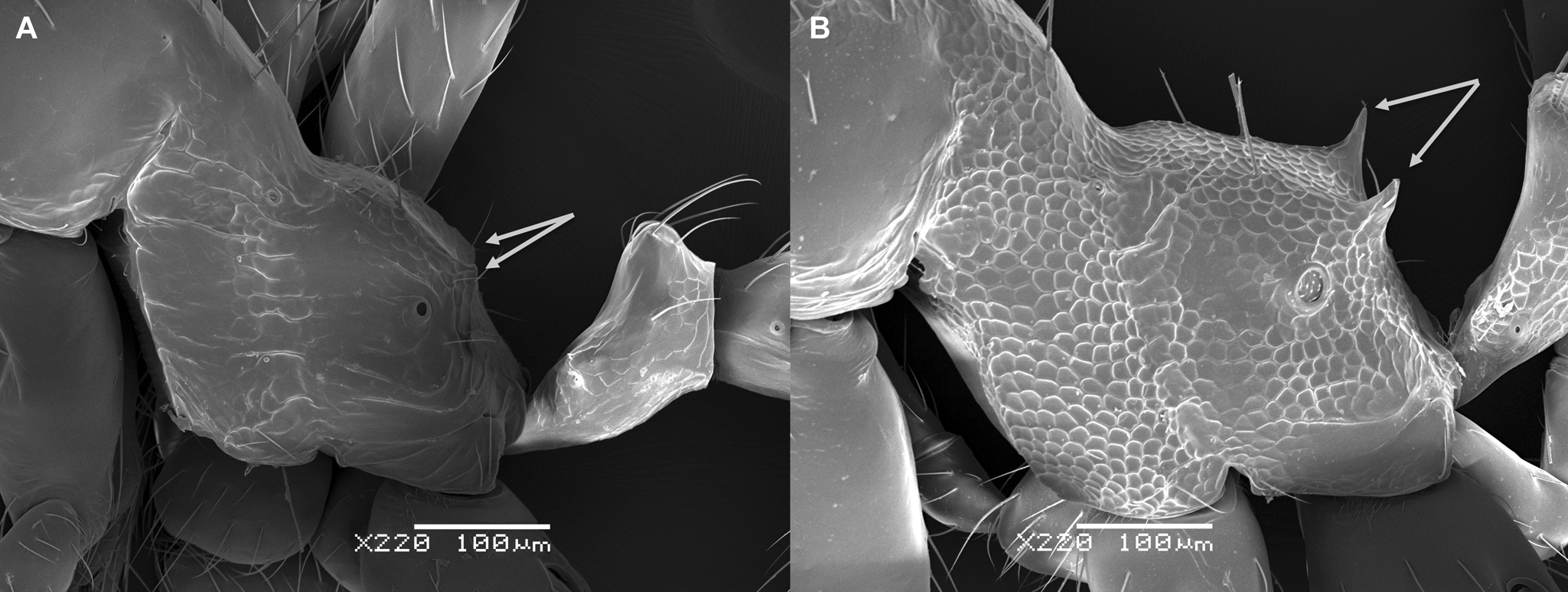

Supplement: Figure S2 — Lateral views of the propodeum showing shorter spines in P. megacephala var 1. than in P. megacephala var. 2 (B). Magnification and scale bar are indicated for each scanning electron microscopic image. (TIF) [file pone.0031480.s002.tif]

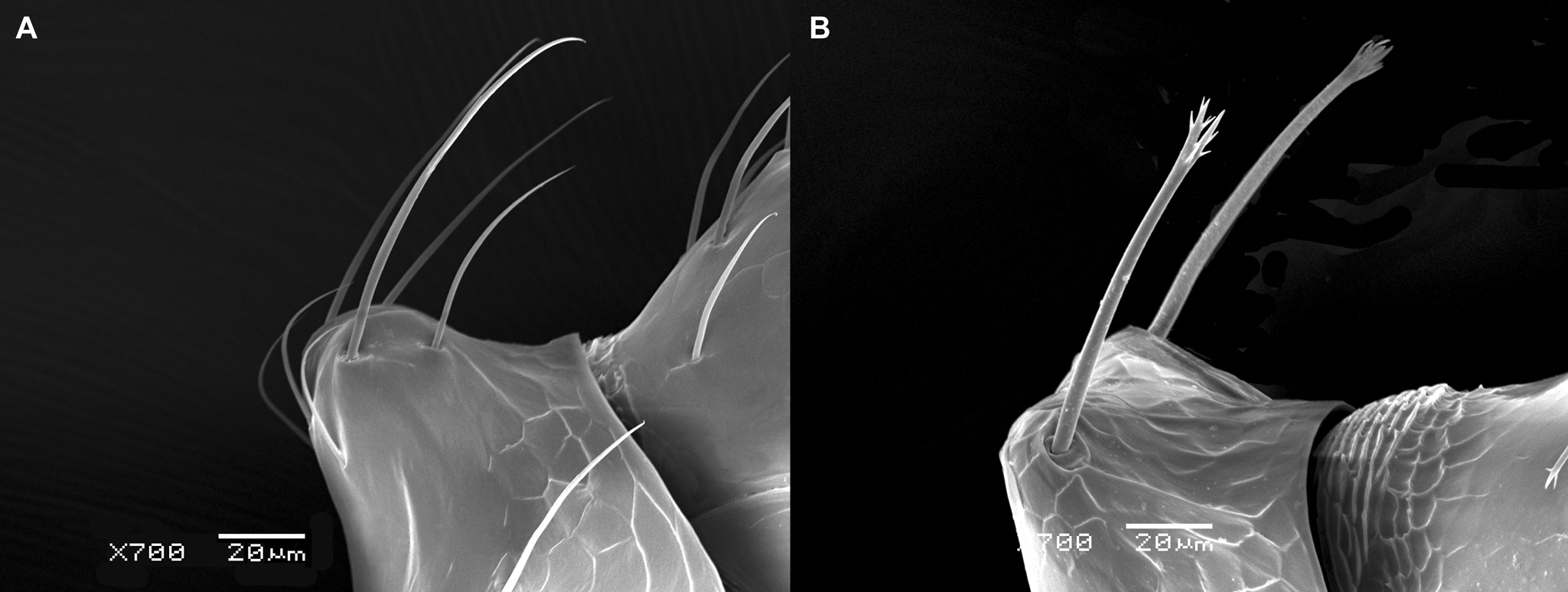

Supplement: Figure S3 — Details in lateral views of hairs present on the petiole. Magnification and scale bar are indicated for each scanning electron microscopic image. The terminal part of the hairs forms a point in P. megacephala var. 1 (A) and a brush in P. megacephala var. 2 (B). (TIF) [file pone.0031480.s003.tif]
